# A cohort study in the assessment of liver fibrosis and markers of disease progression in hepatitis B and D coinfection

**DOI:** 10.1186/1471-2334-13-S1-P53

**Published:** 2013-12-16

**Authors:** Tamer Samir Al Aker, Oana Streinu-Cercel, Anca Streinu-Cercel, Liliana Lucia Preoțescu, Alina Cristina Neguț, Adrian Streinu-Cercel

**Affiliations:** 1 Carol Davila University of Medicine and Pharmacy, Bucharest, Romania; 2 National Institute for Infectious Diseases “Prof. Dr. Matei Balş”, Bucharest, Romania

## Background

Given the important role played by hepatitis viruses in the development of liver fibrosis we consider it necessary to evaluate markers of disease progression and instruments for staging of fibrosis in patients coinfected with hepatitis B and D viruses (HBV, HDV).

## Methods

We performed a cohort study to assess markers of disease progression and staging of liver fibrosis through noninvasive tests at three different time points over the course of HBV and HDV coinfection.

## Results

We assessed data from 64 patients (of which 27 were males - 42%) with a median age of 54±14 (range: 23 to 64 years). The median fibrosis was 0.44±0.23 and the median necroinflammatory activity was 0.54±0.24.

The median FIB-4 score was 1.89±3.23 at the second visit versus 1.26±2.07 at the third visit. The median APRI score was 1.18±1.67 at the second visit compared to 1.79±2.53 at the third visit (p=0.05, 95%CI: 0.0389, 0.43681). Regression analysis showed a correlation between FibroTest (considered the gold standard in this study, in the absence of histology data from liver biopsy), FIB-4 (p=0.43) and APRI (p=0.011).

## Conclusion

Periodic assessment of patients with viral hepatitis is needed, with close monitoring of fibrosis progression and liver function. Noninvasive markers and scores for liver fibrosis are yet to be validated for patients with HBV and HDV coinfection, but the data from this cohort of patients show promising results, with both APRI and FIB-4 positively correlating with FibroTest.

